# Spatial-temporal analysis of cerebral infarction mortality in Hokkaido, Japan: an ecological study using a conditional autoregressive model

**DOI:** 10.1186/s12942-022-00316-1

**Published:** 2022-10-31

**Authors:** Kazuki Ohashi, Toshiya Osanai, Kensuke Fujiwara, Takumi Tanikawa, Yuji Tani, Soichiro Takamiya, Hirotaka Sato, Yasuhiro Morii, Kyohei Bando, Katsuhiko Ogasawara

**Affiliations:** 1 Faculty of Health Sciences, Hokkaido University, N12-W5, Kita-ku, 060-0812 Sapporo, Japan; 2 Department of Neurosurgery, Faculty of Medicine, Graduate School of Medicine, Hokkaido University, N15-W7, Kita-ku, 060-8638 Sapporo, Japan; 3 Graduate School of Commerce, Otaru University of Commerce, 3-5-21, 047-8501 Midori, Otaru Japan; 4 Faculty of Health Sciences, Hokkaido University of Science, 7-15-4-1, Maeda, Teine-ku, 006-8585 Sapporo, Japan; 5 Department of Medical Informatics and Hospital Management, Asahikawa Medical University, E2-1-1-1, 078-8510 Midorigaoka, Asahikawa Japan; 6 Department of Neurosurgery, Otaru General Hospital, 1-1-1, 047-8550 Wakamatsu, Otaru Japan; 7 Department of Neurosurgery, Kitami Red Cross Hospital, N6-E2, Kitami, 090-8666 Sapporo, Japan; 8 Center for Outcomes Research and Economic Evaluation for Health, National Institute of Public Health, 2-3-6, 351-0197 Wako, Minami Japan; 9 Graduate school of Health Sciences, Hokkaido University, N12-W5, Kita-ku, 060-0812 Sapporo, Japan

**Keywords:** Conditional autoregressive model, Cerebral infarction, Bayesian inference, Spatial-temporal analysis, Stroke, Healthcare accessibility

## Abstract

**Background:**

Accessibility to stroke treatments is a challenge that depends on the place of residence. However, recent advances in medical technology have improved health outcomes. Nevertheless, the geographic heterogeneity of medical resources may increase regional disparities. Therefore, evaluating spatial and temporal influences of the medical system on regional outcomes and advanced treatment of cerebral infarction are important from a health policy perspective. This spatial and temporal study aims to identify factors associated with mortality and to clarify regional disparities in cerebral infarction mortality at municipality level.

**Methods:**

This ecological study used public data between 2010 and 2020 from municipalities in Hokkaido, Japan. We applied spatial and temporal condition autoregression analysis in a Bayesian setting, with inference based on the Markov chain Monte Carlo simulation. The response variable was the number of deaths due to cerebral infarction (ICD-10 code: I63). The explanatory variables were healthcare accessibility and socioeconomic status.

**Results:**

The large number of emergency hospitals per 10,000 people (relative risk (RR) = 0.906, credible interval (Cr) = 0.861 to 0.954) was associated with low mortality. On the other hand, the large number of general hospitals per 10,000 people (RR = 1.123, Cr = 1.068 to 1.178) and longer distance to primary stroke centers (RR = 1.064, Cr = 1.014 to 1.110) were associated with high mortality. The standardized mortality ratio decreased from 2010 to 2020 in Hokkaido by approximately 44%. Regional disparity in mortality remained at the same level from 2010 to 2015, after which it narrowed by approximately 5% to 2020. After mapping, we identified municipalities with high mortality rates that emerged in Hokkaido’s central and northeastern parts.

**Conclusion:**

Cerebral infarction mortality rates and the disparity in Hokkaido improved during the study period (2010–2020). This study emphasized that healthcare accessibility through places such as emergency hospitals and primary stroke centers was important in determining cerebral infarction mortality at the municipality level. In addition, this study identified municipalities with high mortality rates that require healthcare policy changes. The impact of socioeconomic factors on stroke is a global challenge, and improving access to healthcare may reduce disparities in outcomes.

**Supplementary information:**

The online version contains supplementary material available at 10.1186/s12942-022-00316-1.

## Background

Mortality rates of cerebral infarction from 2000 to 2019 have improved in Japan [1], with recombinant tissue-type plasminogen activator (rt-PA) therapy and mechanical thrombectomy contributing to this outcome [2–4]. However, these treatments are not widely available [5]. Accessibility to stroke treatments is a challenge that depends on the region in Japan [5]. In other words, advanced treatments have improved the outcome of cerebral infarction nationwide; however, these might expand regional disparities. In studies on the relationship between mortality and medical resources or geographical accessibility to healthcare, Saijo et al. revealed that municipalities with a small distance to primary care were related to low stroke mortality in Hokkaido [6]. Additionally, Kawaguchi et al. reported a negative correlation between the number of institutions with coronary computed tomography angiography and mortality from acute myocardial infarction in Japan [7]. A cohort study in Switzerland clarified that patients with a longer travel time to a high-quality acute care hospital had a high mortality rate due to cerebral infarction and acute myocardial infarction [8]. In addition, poor accessibility to intravenous thrombolysis and endovascular therapy in rural areas has resulted in poor outcomes for stroke in the United States (US) [9]. To summarize, in both of the individual and regional levels, poor access to advanced stroke care is directly related to the clinical outcome of strokes. Heterogeneity in medical resources and accessibility cause regional disparities in stroke mortality.

Hokkaido has the largest area and the lowest population density in Japan. Further, it has a high heterogeneity among urban and rural areas in the medical care system for stroke [10]. Heterogeneity in advanced stroke care may cause regional disparities in clinical outcomes for stroke in Hokkaido. Spatial and temporal analyses can identify health outcome of regional disparities, such as mortality [11]. They can be used to evaluate the relationship between accessibility to advanced stroke care and clinical outcomes for spatio temporal trends. However, the effect of regional disparities in access to stroke care and other related factors on the clinical outcomes of cerebral infarction has not been clarified. Therefore, this spatial and temporal study analyzed the relationship between regional disparities and healthcare accessibility and revealed the trend of cerebral infarction mortality at the municipality level.

## Methods

### Study design

This ecological study used public data from 2010 to 2020. It aimed to clarify the time trend and regional disparity in cerebral infarction mortality for the entire region and locally and to investigate the factors leading to disparity in mortality. The variable under investigation was the number of deaths due to cerebral infarction (ICD-10 code I63) recorded in 188 municipalities in Hokkaido. The standardized mortality ratio (SMR), which is calculated as the ratio between the observed and expected cases, is commonly used to compare the relative mortality risk; however, the SMR is unstable in small populations. To address this, we followed a previous study [12] and employed a spatial and temporal analysis using a conditional autoregressive model and assuming a discrete Poisson distribution to estimate the observed cases.

### Study area

The study area was 188 municipalities in Hokkaido, Japan. Hokkaido is the largest (83,424 km^2^) and northernmost prefecture in Japan with a population of 5.22 million and a population density of 66.6 persons/km^2^. Based on the data of these 188 municipalities, the median population size was 5,795 individuals (range 706 to 329,306)whereas the median area was 367 km^2^ (range 24 to 1,427) (2020) [13].

## Response and explanatory variables

The response variable in this study was the number of deaths due to cerebral infarction in municipalities. Explanatory variables associated with healthcare accessibility were: the number of physicians, general hospitals, clinics, and emergency hospitals per 10,000 population; direct distances between representative points in municipalities and primary stroke centers (PSC) were employed as the proxy variables of geographical accessibility to advanced stroke care. A model included the number of expected deaths as an offset term to adjust for the population size of each municipality. Moreover, in terms of municipality-level socioeconomic status variables, this study adopted; the ratio of people who completed college and university studies (%), the proportion of workers in primary industries (%), such as agriculture and fishing, the proportion of workers in secondary industries (%), such as manufacturing and construction, and the proportion of workers in tertiary industries (%), mainly services [14–17]. However, these socioeconomic values were fixed throughout the study period; therefore, the study only captured the spatial variation in mortality. Of the 41 facilities certified as PSCs by the Japan Stroke Society as of 2021 [18] and Hokkaido University Hospital, the facilities included in the shapefile data [19] of 2010, 2014, and 2020 were defined as PSCs. As a result, this study adopted 39 facilities in 2010–2013 and 2014–2019 as well as 41 facilities in 2020. This study set the addresses of municipal offices in 2010 as a representative point in the municipalities. To calculate reference mortality for cerebral infarction, this study used the mean Hokkaido population and the mean number of deaths due to cerebral infarction from 2010 to 2020 in Hokkaido, with each population stratified by gender and five-year age group (e.g. 0–4, 80 years or older, etc.). The aforementioned mortality was applied to the population of each municipality for each year to calculate the number of expected deaths. Public data and shapefiles were obtained from e-stat [20] and the National Land Numerical Information Download Service [19] (Table 1). Data from the previous survey were used for missing data in case the survey was not conducted.

**Table 1:** Data sources

Data	Source	Year
Population (annual)	Surveys of Population, Population Change and the Number of Households based on the Basic Resident Registration [21]	2010–2020
Number of deaths by cerebral infarction (annual)	Vital statistics [22]	2010–2020
Number of physicians	Statistics of Physicians, Dentists, and Pharmacists [23]	2010, 2012, 2014, 2016, 2018
Number of clinics, general hospitals, and emergency hospitals	Survey of Medical Institutions [24]	2010–2019
Ratio of people who completed college and university studies	Census [13]	2010
Proportion of primary, secondary, and tertiary industries	Census [13]	2015
Polygon data of municipalities	National Land Numerical Information Download Service [19]	2021
Point data of hospitals	National Land Numerical Information Download Service [19]	2010, 2014, 2020
Point data of municipal offices	National Land Numerical Information Download Service [19]	2010

## Statistical analysis

To determine which explanatory variables should be included in the analysis, we applied a non-spatial generalized linear Poisson model, as represented by equation (a), and eliminated one variable when the variance inflation factor was > 5 to avoid multicollinearity.

(a)

$${O_{it}} \sim Poisson({\pi _{it}})$$

$$log{\mu }_{it}=offset\left(log{E}_{it}\right)+a+{\beta X}_{it}+{\psi }_{it}$$

where $i,t$ are municipalities $i$ and year$t$, $O$ is the number of deaths due to cerebral infarction, $\mu$ is the relative mortality risk of cerebral infarction, $E$ is the number of expected deaths, $a$ is the intercept, $\beta$ is the coefficient of an explanatory variable $X$, and $\psi$ is a random effect.

After process of equation (a), we expanded it to the spatial-temporal conditional autoregressive (CAR) model in detail below (b),

(b)

$${O_{it}} \sim Poisson({\pi _{it}})$$

$$log{\mu }_{it}=offset\left(log{E}_{it}\right)+a+{\beta X}_{it}+{\psi }_{it}$$

$${\psi }_{it}={\phi }_{it}$$

$$\left. {{\varphi _t}} \right|{\varphi _{t - 1}} \sim N\left( {{\rho _T}{\varphi _{t - 1}},{\tau ^2}Q{{\left( {W,{\rho _s}} \right)}^{ - 1}}} \right)$$

$${\varphi _1} \sim N\left( {0,{\tau ^2}Q{{\left( {W,{\rho _s}} \right)}^{ - 1}}} \right)$$

$${\tau ^2} \sim Inverse - Gamma\left( {1,0.01} \right)$$

$${\rho _s},{\rho _T} \sim Uniform\left( {0,1} \right)$$

where ${\phi }_{it}$ is a random effect in municipality $i$ and year $t$, $W$ is the spatial adjacency matrix, ${\rho }_{S}$ is the spatial autocorrelation parameter, and ${\rho }_{T}$ is the temporal autocorrelation parameter. ${\rho }_{S}$ and ${\rho }_{T}$ controlled the spatial and temporal autocorrelation, respectively, with a value of 0 corresponding to independence while a value of 1 corresponding to strong autocorrelation. ${\tau }^{2}$ is the variance of the spatial and temporal processes. The precision matrix $Q\left(W, {\rho }_{S}\right)$, introduced by Leroux et al., corresponds to the CAR model [25]. ${\tau }^{2}$, ${\rho }_{S}$, and ${\rho }_{T}$ were assigned an inverse-gamma prior distribution (1, 0.01) and uniform prior distribution (0, 1), respectively. Rushworth et al. introduced this model at first [26], and Lee et al. implemented it as R package “CARbayesST” [11, 27]. Models 1 and 2 were implemented using the function ST.CARar () in the R-package [27]. Model 1 included all variables. Model 2 excluded the ratio of people who completed college and university studies and the proportion of workers in secondary industries and tertiary industries. Model 2 was used to assess the impact of socioeconomic factors on medical resource factors. The models were implemented in a Bayesian setting, with inference based on the Markov chain Monte Carlo (MCMC) simulation. Further, they were estimated using 15,000 samples based on three independent chains and 5,20,000 samples with a burn-in of 20,000 samples thinning to every 100th sample. The convergence of the MCMC simulation was evaluated by inspection of trace plots and Gelman–Rubin’s potential scale reduction factor (< 1.1) [28]. The spatial adjacency matrix was defined based on the queen method, and island municipalities were considered adjacent to a municipality if a ferry line connected them i.e., if the municipalities share da border and ferry line, they received a value of 1. Otherwise, they were assigned a value of 0. In addition, we ran Moran’s I with 9,999 permutations on the original SMR and residuals after analysis to check for the effects of autocorrelation.

Our results demonstrated the posterior median of the coefficients and 95% credible interval (Cr) in relative risk (RR) per one standard deviation for explanatory variables. Moreover, this study showed the trend of modeled SMR obtained by the number of deaths with posterior estimation as well as regional disparity of modeled SMR by the difference in interquartile range (IQR) [27, 29], changes of modeled SMR from 2010 to 2020, and posterior exceedance probabilities (modeled SMR > 1.0).

To examine the influence of the priors on the estimation of the posterior distribution, a sensitivity analysis was performed with an inverse gamma distribution (0.5, 0.005) and (1, 0.05) to ${\tau }^{2}$ in Model 1. ArcGIS 2.8 (ESRI, Redlands, CA, USA, https://www.esri.com/en-us/home) measured the distance between municipalities and PSC, and all other analyses were performed using R (version 4.1.1) [30].

## Results

The total number of deaths due to cerebral infarction was 31,510, ranging from 2,622 to 3,118 per year from 2010 to 2020. The number of deaths per year decreased in Hokkaido; however, approximately 0–200 people died annually in each municipality (Table 2).

**Table 2:** The trend in number of deaths by cerebral infarction in Hokkaido, 2010–2020

	Total	Median	Range	Population
2010	3118	5	0-186	5,520,894
2011	3139	6	0-230	5,498,916
2012	3003	6	0-219	5,474,216
2013	2983	6	0-155	5,462,664
2014	2914	5	0-204	5,460,246
2015	2812	5	0-200	5,429,222
2016	2766	5	0-204	5,388,911
2017	2759	5	0-205	5,368,540
2018	2726	5	0-233	5,337,149
2019	2668	5	0-188	5,302,163
2020	2622	4	0-212	5,265,727

Median expressed the median number of deaths in municipalities.Data source: e-stat [20]

## Exploratory analysis by non-spatial generalized linear poisson model

As a result of exploring explanatory variables, the following eight variables were employed: the number of physicians, general hospitals, clinics, and emergency hospitals per 10,000 population; distance to PSC; the ratio of people who completed college and university studies; the proportion of workers in secondary industries (%); and the proportion of workers in tertiary industries (%). Table 3 shows that the number of emergency hospitals per 10,000 people (β =-0.131) and the ratio of people who completed college and university studies (β ＝-0.717) were related to low mortality. In contrast, the number of general hospitals (β ＝0.148), the number of clinics (β＝0.006), the distance to PSC (β ＝0.001), and the proportion of workers in secondary industries (β＝1.165) were related to high mortality.

**Table 3:** Results of exploratory analysis by the non-spatial generalized Poisson model

Variables	Coefficient	95% CI	z-score	VIF
Intercept	-0.237	-0.384, -0.092	-3.192	
Physicians (per 10,000)	0.001	-0.000, 0.002	1.150	2.322
General hospitals (per 10,000)	0.148	0.118, 0.176	9.719	2.389
Clinics (per 10,000)	0.006	0.001, 0.011	2.313	1.480
Distance to PSC (km)	0.001	0.001, 0.002	4.304	1.711
Emergency hospitals (per 10,000)	-0.131	-0.166, -0.096	-7.296	2.496
Ratio of people who completed college and university studies (%)	-0.717	-1.179, -0.254	-3.037	2.958
Proportion of workers in secondary industries (%)	1.165	0.869, 1.460	7.726	1.607
Proportion of workers in tertiary industries (%)	-0.043	-0.234, 0.148	-0.443	2.412

CI; Confidence interval, VIF; Variance inflation factor, PSC; Primary stroke center

## Spatial and temporal CAR model

Model 1 included seven variables (healthcare accessibility and socioeconomic status) after exploratory analysis, while Model 2 handled five variables, excluding socioeconomic status. The results of Model 1 in Table 4 showed that the variable of “emergency hospitals” (RR = 0.905), per one standard deviation (SD), reduced the mortality by 9.4%. On the other hand, the variables “general hospitals” (RR = 1.124) and “distance to PSC” (RR = 1.062) increased the mortality per one SD by 12.4% and 6.2%, respectively. The 95% Cr of RR of other five variables included one. In Model 2, all parameters were similar to Model 1 thus the healthcare accessibility variables were robustness to the results. As a result, we adopted the Model 1 for mapping for further analysis. The convergence of MCMC simulation in Model 1 was confirmed by a trace plot and a potential scale reduction factor (< 1.1) (Additional File 1). We examined the trace plots for some area modelled SMRs in each year. Sub-sample of fitted value trace plots were provided in Additional File 1.

**Table 4:** Relationship relative risk and explanatory variables

	Model 1		Model 2		1SD
	RR	95% Cr	RR	95% Cr	
Physicians (per 10,000)	1.009	0.989, 1.029	1.001	0.983, 1.020	10.22
General hospitals (per 10,000)	1.123	1.068, 1.178	1.110	1.058, 1.164	1.12
Clinics (per 10,000)	0.998	0.963, 1.033	0.984	0.950, 1.019	4.47
Distance to PSC (km)	1.061	1.014, 1.110	1.067	1.024, 1.113	30.64
Emergency hospitals (per 10,000)	0.906	0.861, 0.954	0.913	0.868, 0.960	1.03
Ratio of people who completed college and university studies	0.964	0.927, 1.000	-	-	0.03
Proportion of workers in secondary industries	1.030	0.998, 1.063	-	-	0.06
Proportion of workers in tertiary industries	1.012	0.980, 1.047	-	-	0.11

Model 1 contained all variables. Model 2 excluded the ratio of people who completed college and university studies, the proportion of workers in secondary industries, and the proportion of workers in tertiary industries. RR; Relative risk, Cr; Credible interval, SD; Standard deviation, PSC; Primary stroke center

Table 5 showed that Model 1 had appropriateness to manage for spatial autocorrelation. There is no significant spatial autocorrelation of the residuals of model 1 in all years.

**Table 5:** Results of Moran’s I test for original SMR and residuals after Model 1

	SMR	Residuals (chain 1)
2010	-0.03	-0.10
2011	-0.00	-0.03
2012	0.08*	-0.00
2013	0.08*	-0.03
2014	0.06	-0.10
2015	-0.03	-0.20
2016	0.13**	-0.14
2017	-0.02	-0.05
2018	0.10*	-0.07
2019	0.05	-0.07
2020	0.11**	0.06

SMR; Standardized mortality ratio, **p-value < 0.01, * p-value < 0.05

## Sensitivity analysis

The sensitivity analysis results obtained by using two distinct priors revealed the same spatial pattern and parameter results as Model 1 (Additional File 2). Therefore, the effect of priors on the parameter was less noticeable. This indicated that the prior probabilities were adequate in Model 1.

## Global and local trend analysis of modeled SMR in Hokkaido, 2010–2020

**Fig. 1:**
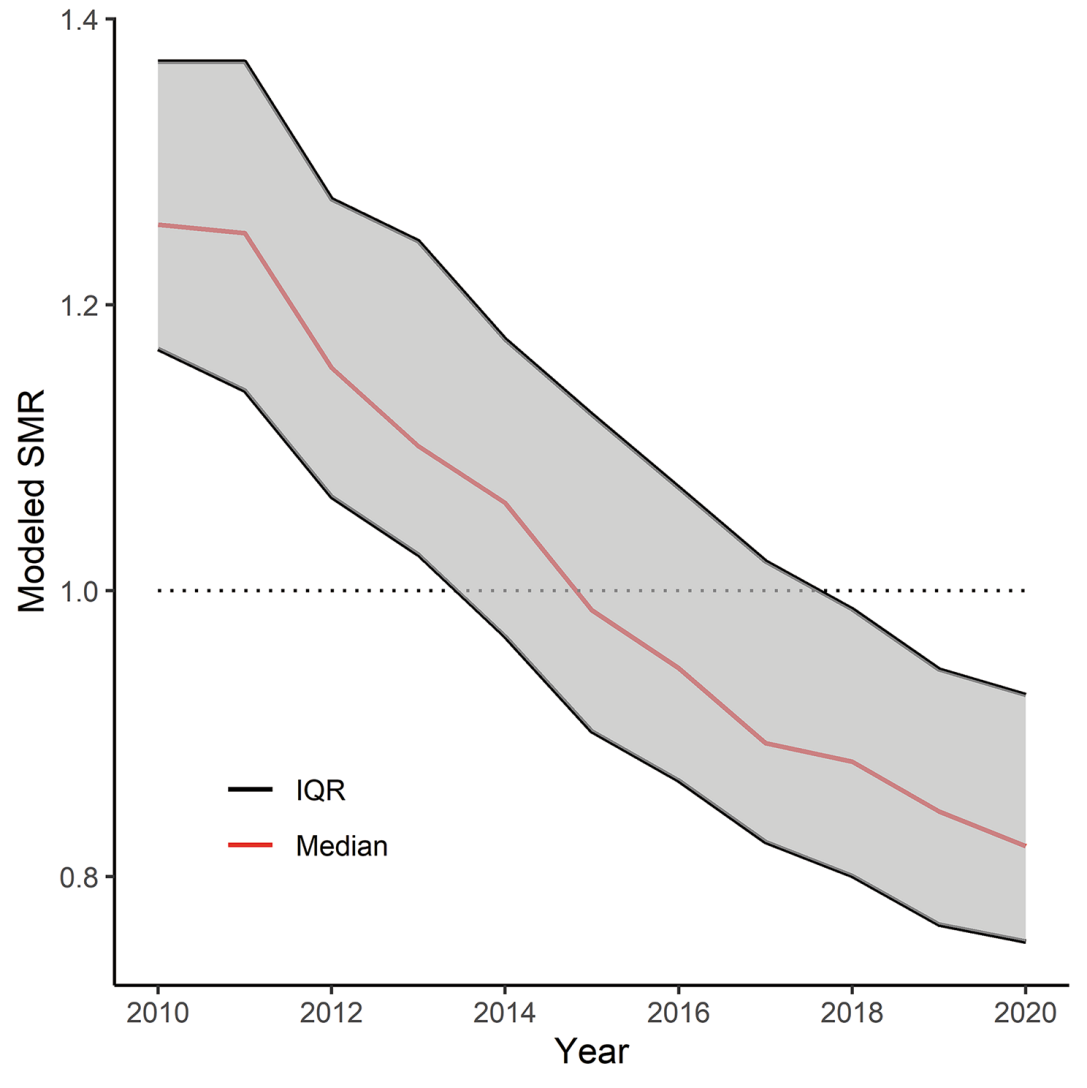
Trend of modeled SMR, 2010–2020

Figure 1 showed that the modeled SMR decreased continuously from 2010 to 2020 in Hokkaido. Moreover, regional disparity, which was evaluated for differences of IQR, fluctuated between approximately 20% and 23% for the period of 2010–2015, after which it narrowed by approximately 5% for the consequent period of 2015–2020. In summary, cerebral infarction mortality and regional disparities improved during the study period in Hokkaido—cerebral infarction mortality decreased by approximately 44% over 11 years.

**Fig. 2:**
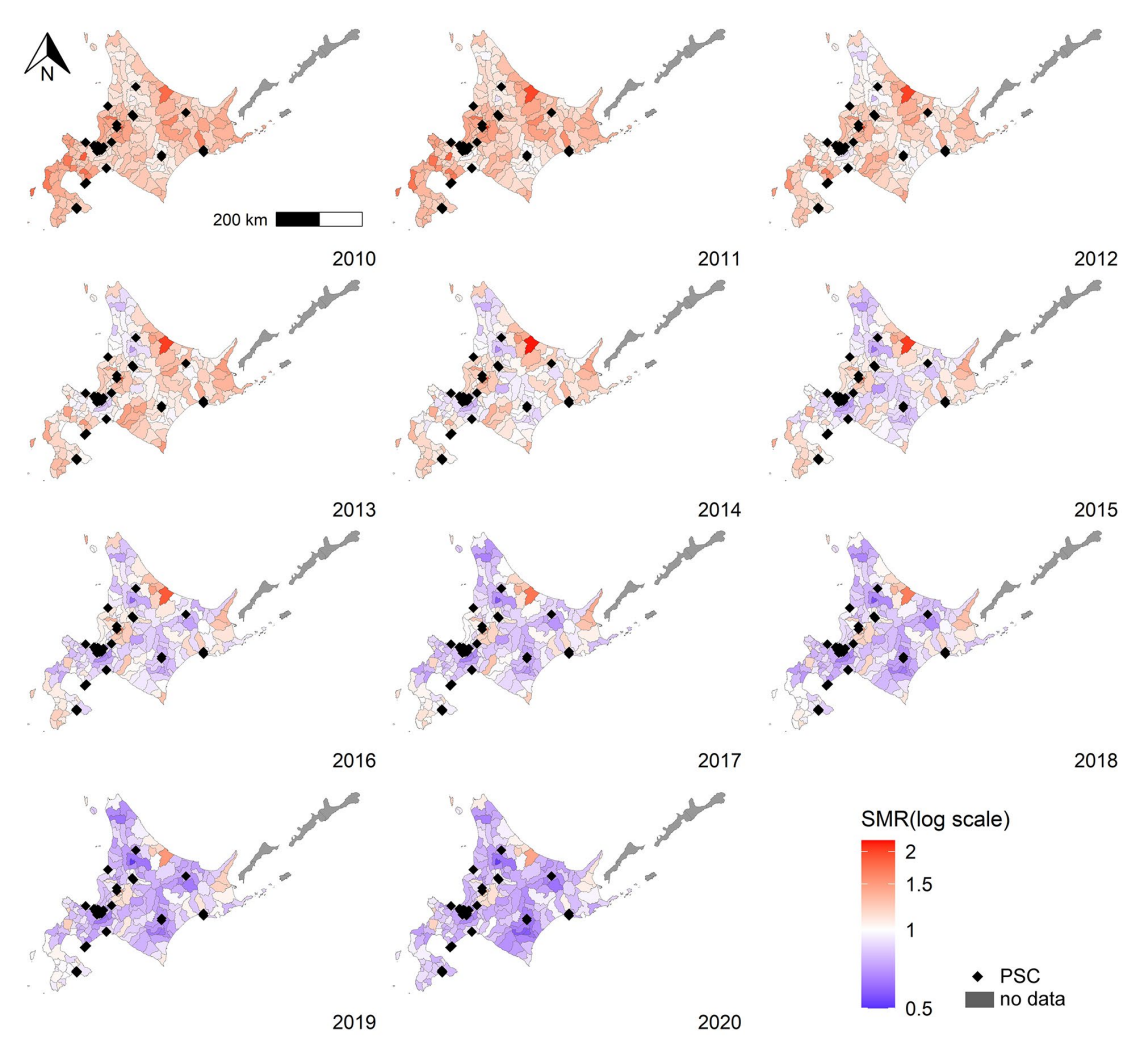
Map of modeled SMR, 2010–2020. PSC, primary stroke center; SMR, standardized mortality ratio. The white area represents the modeled SMR 1. The red area indicates the municipalities with a modeled SMR > 1, and the blue area indicates the municipalities with a modeled SMR < 1

Figure 2 showed that municipalities with high mortality rates emerged in the center and northeast throughout 2010–2020. The dark red area indicates high mortality, and the dark blue area indicates low mortality. The white color area represented that modeled SMR was one as reference mortality rate throughout study period. This spatial pattern meant that mortality was declining in almost every area.

**Fig. 3:**
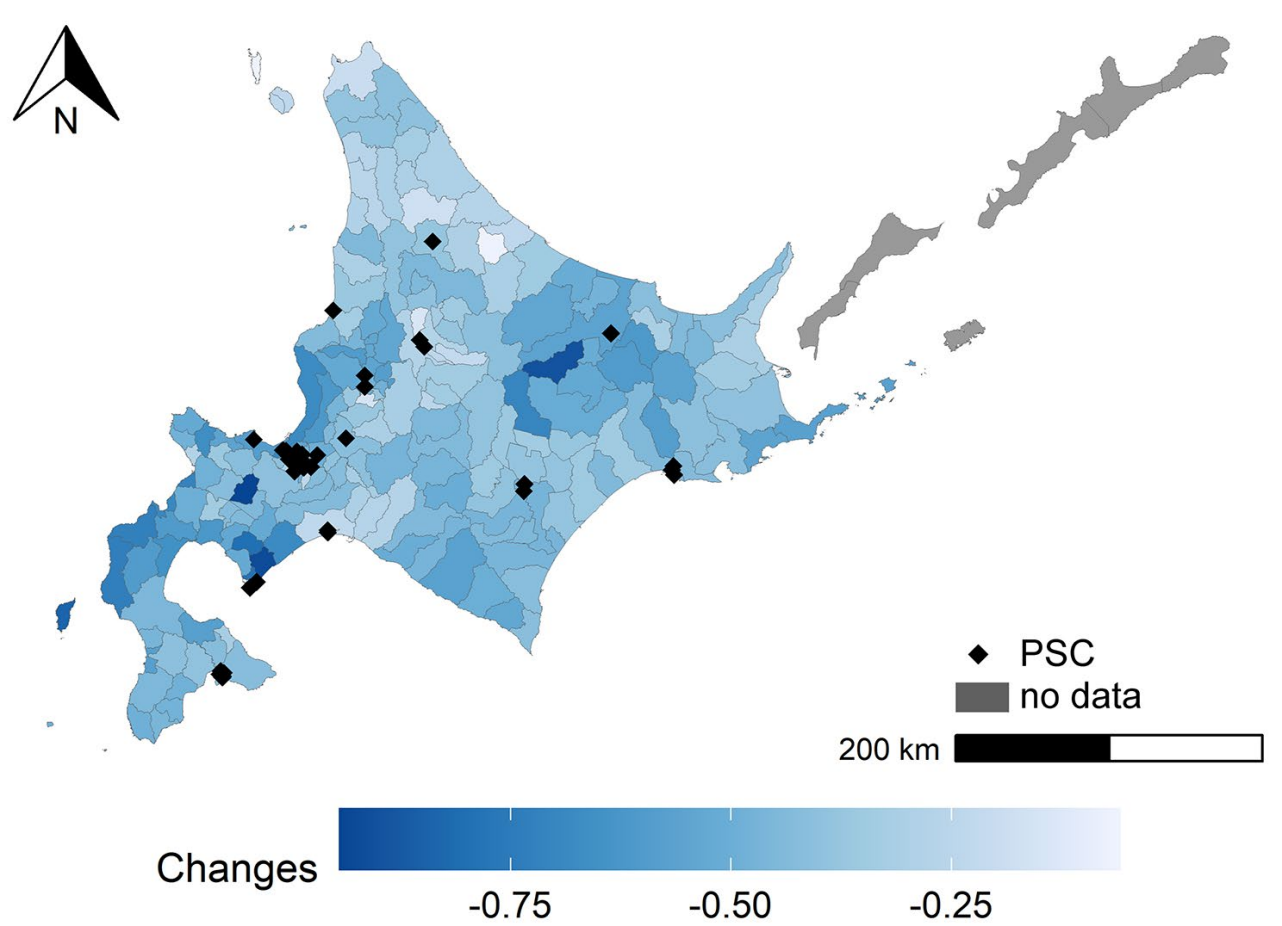
Difference in modeled SMR from 2010 to 2020. PSC, primary stroke center. Darker blue indicates a large decrease in modeled SMR

Figure 3 showed the difference in modeled SMR between 2010 and 2020. The light blue area shows little change in modeled SMR, whereas the dark blue area shows a large change in modeled SMR. The mean difference in modeled SMR was − 0.456 in 2020. The blue areas stand out in the east and southwest.

**Fig. 4:**
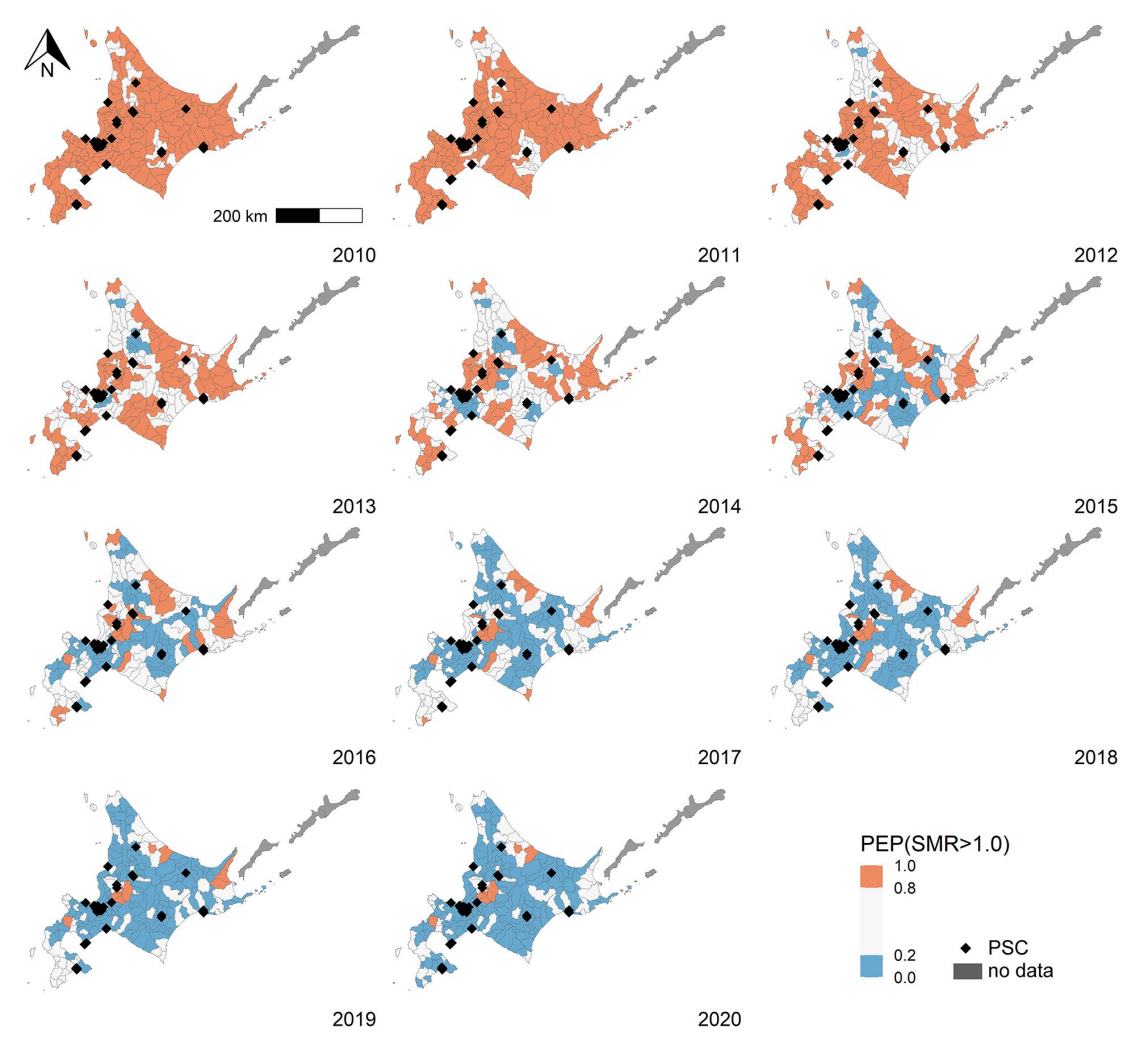
Posterior exceedance probabilities (modeled SMR > 1.0), 2010–2020. PSC, primary stroke center; PEP, posterior exceedance probabilities; SMR, standardized mortality ratio. Red and blue indicates the municipalities with high and low posterior exceedance probability, respectively

Figure 4 showed the posterior exceedance probabilities (modeled SMR > 1.0) in municipalities across 2010–2020. The red area indicated a high probability (0.8 to 1.0) of a modeled SMR > 1.0. The blue area indicated a low probability (0.0 to 0.2) of a model. This map expressed the spatial pattern similar to Fig. 2; particularly, it emphasized municipalities with high and low mortality, respectively.

## Discussion

### Time trend of cerebral infarction mortality and risk factors in Hokkaido

In Hokkaido, the mortality rate due to cerebral infarction decreased by approximately 44% from 2010 to 2020, and the regional disparity in mortality due to cerebral infarction narrowed after 2015. This study is the first to investigate cerebral infarction mortality at the municipal level in Japan using spatial and temporal CAR models. This decreasing trend in Hokkaido was similar to the results of a registry study targeting Japan by Toyoda et al. [1]. Regarding the relationship between mortality and healthcare accessibility, mortality was low when there were many emergency hospitals per 10,000 people. Regarding distance to PSC, increased distance was associated with higher mortality with an approximately 6% increase in mortality when the direct distance was extended by approximately 30 km. Thus, poor healthcare accessibility may be related to increased mortality. The above cohort study revealed that longer driving times to central and university hospitals increased stroke mortality in the population aged ≥ 65 years [8]. This is partially in agreement with our results. Given that some emergency hospitals are also PSCs, the mortality from cerebral infarction decreases in areas where advanced care such as rt-PA therapy and mechanical thrombectomy are readily available as initial treatments at the onset of cerebral infarction. The American Stroke Association has stated the importance of screening for strokes as pre-hospital care as well as rapid transportation to the highest level stroke care for the treatment of cerebral infarctions [31, 32]. Therefore, ensuring transportation to emergency hospitals and effective initial treatment is essential for cerebral infarction care. These findings emphasize the improvement of geographical accessibility by the cooperative method [33] and optimizing the location of PSCs [10, 34]. In contrast, a large number of general hospitals were associated with high mortality. This study confirmed robustness of healthcare variable for socioeconomic status by running Model 2 excluded the socioeconomic variables. Both models showed that the number of general hospitals was positively associated with mortality. One of the reasons for this result may be related to the lack of specialized departments in general hospitals. Many general hospitals in this study do not have a department of neurosurgery. It means that those hospitals do not provide more effective treatments for an acute cerebral infarction. However, these do not provide a basis for understanding the positive relationship with mortality. To explain this relationship, further research required.

### Regional disparities in cerebral infarction mortality

The Law on Stroke and Cardiovascular Disease enacted in 2018 promoted the development of appropriate medical systems for cardiovascular diseases, including stroke [35]. One of the goals of the law is to improve cardiovascular disease outcomes by ensuring the quality of advanced medical care nationwide. Figure 3 shows municipalities with reduced mortality for cerebral infarction around PSCs and provides evidence of the benefits of securing medical care systems. Distance to PSC was a variable that was significantly associated with mortality, and this result was consistent with previous studies on the relationship between good access to acute care and low mortality [7, 8]. However, mortality was not reduced in some municipalities, even though there was a PSC in the area. Simultaneously, Figs. 2 and 4 showed that municipalities with high mortality rates emerged in Hokkaido in 2020. As a limitation of this analysis, it is natural that the mortality rate does not improve relatively in the municipalities with low mortality rate as of 2010. Another possible explanation for this result is that the functioning of the PSCs defined in the study is not equal across all study periods. There is a mix of PSC that were fully functioning early on and those that only began functioning in recent years. In fact, although not observed, there were differences in performance among PSCs over the study period. This is explained by the heterogeneity of stroke care resources in Hokkaido [10]. In the US and Japan, some studies have reported regional disparities between urban and rural areas in rt-PA therapy and mechanical thrombectomy [5, 36]. Moreover, mortality was 1.11 times greater in rural areas than that in urban areas in the US because of poor access to emergency medicine [37]. However, this study did not handle the actual medical results of each PSC or emergency hospital and did not determine the supply and demand for advanced stroke care in the area. This study identified municipalities with high mortality rates, regardless of whether they were considered urban or rural areas. Municipalities with high mortality rates may have low accessibility to emergency hospitals and PSCs, thus requiring healthcare policy implementations to improve mortality rates. Therefore, future investigations are required to determine whether the medical system is functioning properly and whether staffing and equipment shortages occur.

### Strengths and limitations

This study investigated cerebral infarction mortality between cities, wards, towns, and villages of different populations using spatial and temporal CAR models [27] and identified municipalities with high-risk mortality. The strength of this method is that it stabilizes the SMR of municipalities with small populations. Moreover, this study showed the importance of how access to healthcare affects cerebral infarction outcome after adjusting for socioeconomic factors at the municipality level. Although the impact of individual and regional socioeconomic factors on stroke is a global challenge [38, 39], improving access to healthcare (i.e., emergency hospital or acute treatment for cerebral infarction) may reduce disparities in outcomes caused by differences in socioeconomic status. However, this study has some limitations. First, the direct distance between the representative points of municipalities and PSCs was used as a proxy variable for geographical accessibility. This was the simplest assumption as it presumed that patients would move to the same PSC from any location in the municipality. This assumption had no considerations such as road distance, driving time, and differences in locations of the municipality. Using more precise road distances and driving times instead of direct distance, as well as variables such as demand and supply, could affect the results. However, direct distance also has the advantage of averaging out the differences in transportation, such as air ambulance and on-road ambulance, as well as changes in weather and traffic conditions, which makes it easy to measure over time. Second, we did not guarantee the availability of previous PSCs—all PSCs are based on the current information. In other words, it may overestimate access to PSCs in some areas.

Third, this study did not handle the number of rt-PA therapies and mechanical thrombectomy treatment available for each municipality and year. These are crucial treatments that affect stroke outcomes. However, the straight linear distance to the PSC has the meaning of proxy variable for the crucial treatments. Areas closer to the PSC will likely receive those treatments. Finally, an ecological study was conducted without individual data. Thus, the individual risk factors for cerebral infarction (i.e., diabetes and hypertension) were not controlled for. Simultaneously, the impact of these individual risk factors on cerebral infarction mortality at municipality level has not been addressed yet. Future studies using individual data that include the individual risk factors are needed.

## Conclusion

The mortality rate of cerebral infarction steadily decreased in Hokkaido from 2010 to 2020. The regional disparities narrowed after 2015. In the future, improving access to emergency hospitals and PSCs can reduce mortality rates and regional disparity due to cerebral infarction. In addition, this study identified municipalities with high mortality rates that require healthcare policy intervention.

## Electronic supplementary material

Below is the link to the electronic supplementary material.

**Additional File 1Figure S1.** Trace plot of parameters of Model 1; **Table S1.** Potential scale reduction factors of Models 1 and 2 by Gelman-Rubin; **Figure S2. **Trace plot of fitted value in a sub-samples.

**Additional File 2 Figure S3. **Spatial pattern of cerebral infarction mortality by alternative prior (0.5, 0.005); **Figure S4. **Comparison of modeled SMR in three priors; **Table S2.** Comparison of relative risk by Sensitivity analysis for Model 1 using two distinct priors.

## Data Availability

The datasets used and analyzed during the current study are available from the corresponding author upon reasonable request.

## List of abbreviations

rt-PA: Recombinant tissue-type plasminogen activator
SMR: Standardized mortality ratio
PSC: Primary stroke center
CAR: Conditional autoregressive
MCMC: Markov chain Monte Carlo
Cr: Credible interval
RR: Relative risk
SD: Standard deviation
US: United States

## References

[1] Toyoda K, Yoshimura S, Nakai M, Koga M, Sasahara Y, Sonoda K (2022). Twenty-year change in severity and outcome of ischemic and hemorrhagic strokes. JAMA Neurol.

[2] Toyoda K, Koga M, Naganuma M, Shiokawa Y, Nakagawara J, Furui E (2009). Routine use of intravenous low-dose recombinant tissue plasminogen activator in Japanese patients: General outcomes and prognostic factors from the SAMURAI register. Stroke.

[3] Nakagawara J, Minematsu K, Okada Y, Tanahashi N, Nagahiro S, Mori E (2010). Thrombolysis With 0.6 mg/kg intravenous alteplase for Acute ischemic Stroke in Routine Clinical Practice: The Japan post-Marketing Alteplase Registration Study (J-MARS). Stroke.

[4] Goyal M, Menon BK, van Zwam WH, Dippel DWJ, Mitchell PJ, Demchuk AM (2016). Endovascular thrombectomy after large-vessel ischaemic stroke: A meta-analysis of individual patient data from five randomised trials. Lancet.

[5] Maeda M, Fukuda H, Matsuo R, Ago T, Kitazono T, Kamouchi M (2021). Regional disparity of reperfusion therapy for acute ischemic stroke in Japan: A retrospective analysis of nationwide claims data from 2010 to 2015. J Am Heart Assoc.

[6] Saijo Y, Yoshioka E, Kawanishi Y, Nakagi Y, Hanley SJB, Yoshida T (2018). Relationships between road-distance to primary care facilities and ischemic heart disease and stroke mortality in Hokkaido, Japan: A Bayesian hierarchical approach to ecological count data. J Gen Fam Med.

[7] Kawaguchi H, Koike S, Sakurai R, Ohe K (2018). Association between number of institutions with coronary computed tomography angiography and regional mortality ratio of acute myocardial infarction: A nationwide ecological study using a spatial Bayesian model. Int J Health Geogr.

[8] Berlin C, Panczak R, Hasler R, Zwahlen M, Swiss National Cohort Study Group (2016). Do acute myocardial infarction and stroke mortality vary by distance to hospitals in Switzerland? Results from the Swiss National Cohort Study. BMJ Open.

[9] Hammond G, Waken RJ, Johnson DY, Towfighi A, Joynt Maddox KE (2022). Racial inequities across rural strata in acute stroke care and in-hospital mortality: National trends over 6 years. Stroke.

[10] Fujiwara K, Osanai T, Kobayashi E, Tanikawa T, Kazumata K, Tokairin K (2018). Accessibility to Tertiary Stroke Centers in Hokkaido, Japan: Use of novel metrics to assess acute stroke care quality. J Stroke Cerebrovasc Dis.

[11] Lee D, Rushworth A, Napier G. Spatio-temporal areal unit modeling in R with conditional autoregressive priors using the CARBayesST package. J Stat Softw. 2018;84. 10.18637/jss.v084.i09.

[12] Wami W, Walsh D, Hennig BD, McCartney G, Dorling D, Galea S (2021). Spatial and temporal inequalities in mortality in the USA, 1968–2016. Health Place.

[13] Population Census 2010–2020, Ministry of Internal Affairs and Communications. Japan. 2020. https://www.e-stat.go.jp/. Accessed 28 Jul 2022.

[14] Kim Y, Twardzik E, Judd SE, Colabianchi N (2021). Neighborhood socioeconomic status and stroke incidence: A systematic review. Neurology.

[15] Ptushkina V, Seidel-Jacobs E, Maier W, Schipf S, Völzke H, Markus MRP (2021). Educational level, but not income or area deprivation, is related to macrovascular disease: Results from two population-based cohorts in Germany. Int J Public Health.

[16] Zhu Y, Lu Y, Zhou M, Huang P, Zhang P, Guo Y (2021). Occupational class differences in outcomes after ischemic stroke: A prospective observational study. BMC Public Health.

[17] Zaitsu M, Kato S, Kim Y, Takeuchi T, Sato Y, Kobayashi Y (2019). Occupational class and risk of cardiovascular disease incidence in Japan: Nationwide, multicenter, hospital-based case-control study. J Am Heart Assoc.

[18] List of primary stroke center. The Japan Stroke Society. Japan. 2022. https://www.jsts.gr.jp/facility/psc/index.html. Accessed 25 Mar 2022.

[19] Ministry of Land. Infrastructure, Transport and Tourism. National land numerical information download Site. https://nlftp.mlit.go.jp/index.html. Accessed 29 Mar 2022.

[20] Portal Site of Official Statistics of Japan. e-stat. https://www.e-stat.go.jp/en Accessed 29 Mar 2022.

[21] Portal site of official statistics of Japan. Surveys of population, population change and the number of households based on the basic resident registration. https://www.e-stat.go.jp/stat-search/files?page=1&toukei=00200241&tstat=000001039591. Accesssed 29 Mar 2022.

[22] Portal site of official statistics of Japan. Vital statistics. https://www.e-stat.go.jp/en/statistics/00450011. Accessed 29 Mar 2022.

[23] Portal site of official statistics of Japan. Statistics of physicians, dentists and pharmacists. https://www.e-stat.go.jp/en/statistics/00450026. Accessed 29 Mar 2022.

[24] Portal site of official statistics of Japan. Survey of medical institutions. https://www.e-stat.go.jp/en/statistics/00450021. Accessed 29 Mar 2022.

[25] Leroux BG, Lei X, Breslow N. Estimation of Disease Rates in Small Areas: A new Mixed Model for Spatial Dependence. In: Halloran ME, Berry D, editors. Statistical models in epidemiology, the environment, and clinical trials. New York: Springer New York; 2000. p. 179 – 91.

[26] Rushworth A, Lee D, Mitchell R (2014). A spatio-temporal model for estimating the long-term effects of air pollution on respiratory hospital admissions in Greater London. Spat Spatiotemporal Epidemiol.

[27] Lee D (2020). A tutorial on spatio-temporal disease risk modelling in R using Markov chain Monte Carlo simulation and the CARBayesST package. Spat Spatiotemporal Epidemiol.

[28] Gelman A, Carlin JB, Stern HS, Dunson DB, Vehtari A, Rubin DB. Bayesian data analysis. 3rd ed.: Chapman & Hall/CRC; 2013.

[29] World Health Organization [Guideline]. Iron supplementation in postpartum women. https://apps.who.int/iris/handle/10665/249242(2016). Accessed 2 May 2022. 27583315

[30] R Core Team. R: A Language and Environment for Statistical Computing. https://www.r-project.org/. Accessed 25 Mar 2020.

[31] Adeoye O, Nyström KV, Yavagal DR, Luciano J, Nogueira RG, Zorowitz RD (2019). Recommendations for the establishment of stroke systems of care: A 2019 update. Stroke.

[32] Jauch EC, Schwamm LH, Panagos PD, Barbazzeni J, Dickson R, Dunne R, et al. Prehospital stroke system of care consensus conference. Recommendations for Regional Stroke Destination PLANS in Rural, Suburban, and Urban Communities From the Prehospital Stroke System of Care Consensus Conference: A Consensus Statement From the American Academy of Neurology, Am Heart Assoc/American Stroke Association, American Society of Neuroradiology, National Association of EMS Physicians, National Association of State EMS Officials, Society of NeuroInterventional Surgery, and Society of Vascular and Interventional Neurology: Endorsed by the Neurocritical Care Society. Stroke. 2021; doi:10.1161/STROKEAHA.120.033228.

[33] Osanai T, Ito Y, Ushikoshi S, Aoki T, Kawabori M, Fujiwara K (2019). Efficacy of ‘drive and retrieve’ as a cooperative method for prompt endovascular treatment for acute ischemic stroke. J Neurointerv Surg.

[34] Leira EC, Fairchild G, Segre AM, Rushton G, Froehler MT, Polgreen PM (2012). Primary stroke centers should be located using maximal coverage models for optimal access. Stroke.

[35] Kuwabara M, Mori M, Komoto S (2021). Japanese National plan for promotion of measures against cerebrovascular and cardiovascular disease. Circulation.

[36] Gonzales S, Mullen MT, Skolarus L, Thibault DP, Udoeyo U, Willis AW (2017). Progressive rural–urban disparity in acute stroke care. Neurology.

[37] Georgakakos PK, Swanson MB, Ahmed A, Mohr NM (2022). Rural stroke patients have higher mortality: An improvement opportunity for rural emergency medical services systems. J Rural Health.

[38] Marshall IJ, Wang Y, Crichton S, McKevitt C, Rudd AG, Wolfe CD (2015). The effects of socioeconomic status on stroke risk and outcomes. Lancet Neurol.

[39] Yadav RS, Chaudhary D, Avula V, Shahjouei S, Azarpazhooh MR, Abeidi V (2022). Social detaminats of stroke hosptalixation and mortality in United State’s counties. J Clin Med.

