# Supplementary material for: Spatial-temporal analysis of cerebral infarction mortality in Hokkaido, Japan: an ecological study using a conditional autoregressive model
Source: Int J Health Geogr. 2022 Oct 31;21:16. doi: 10.1186/s12942-022-00316-1 (PMC9623919; doi:10.1186/s12942-022-00316-1)

Figure S1. Trace plot of parameters of Model 1.


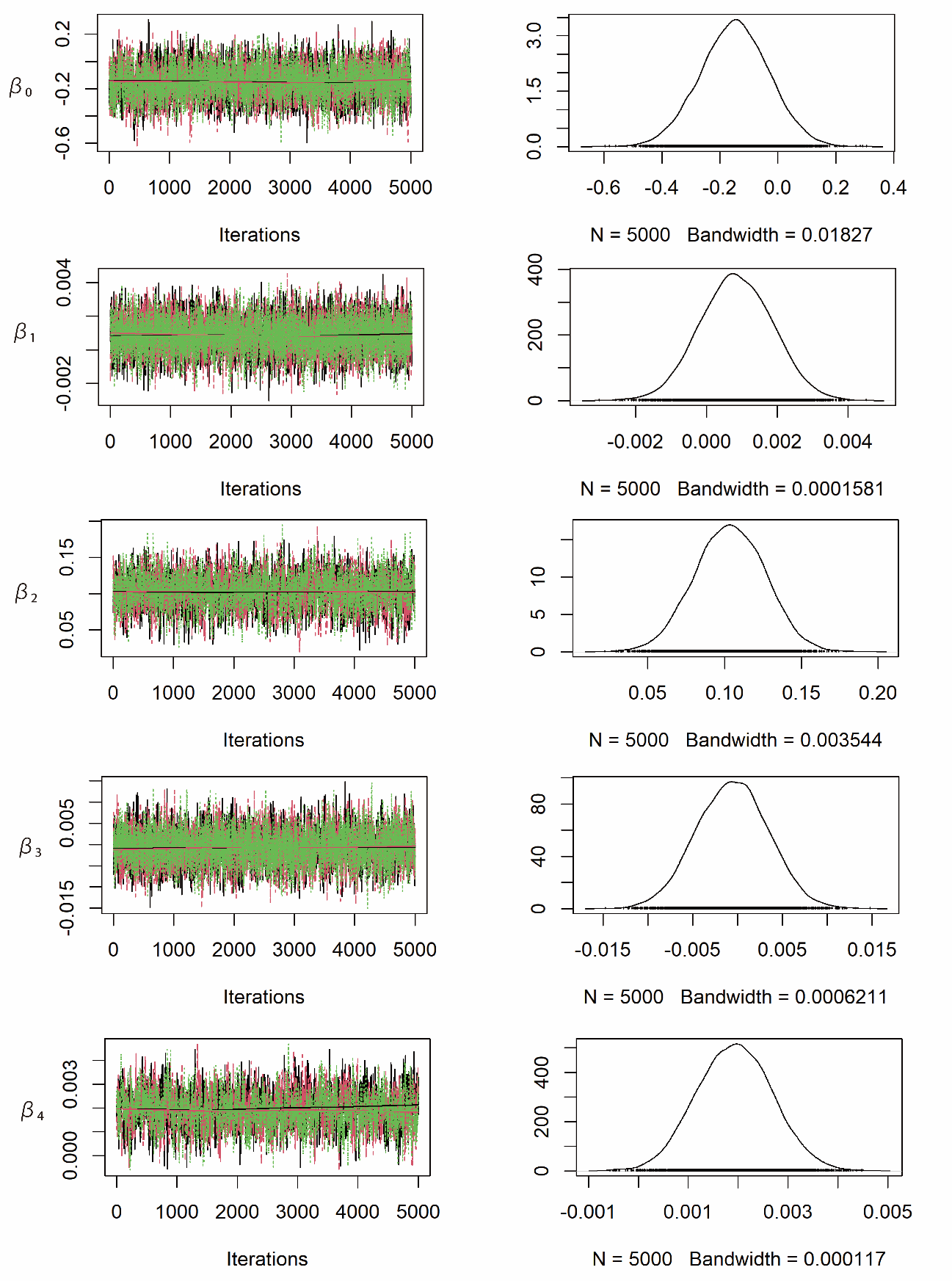


Figure S1 (continued)


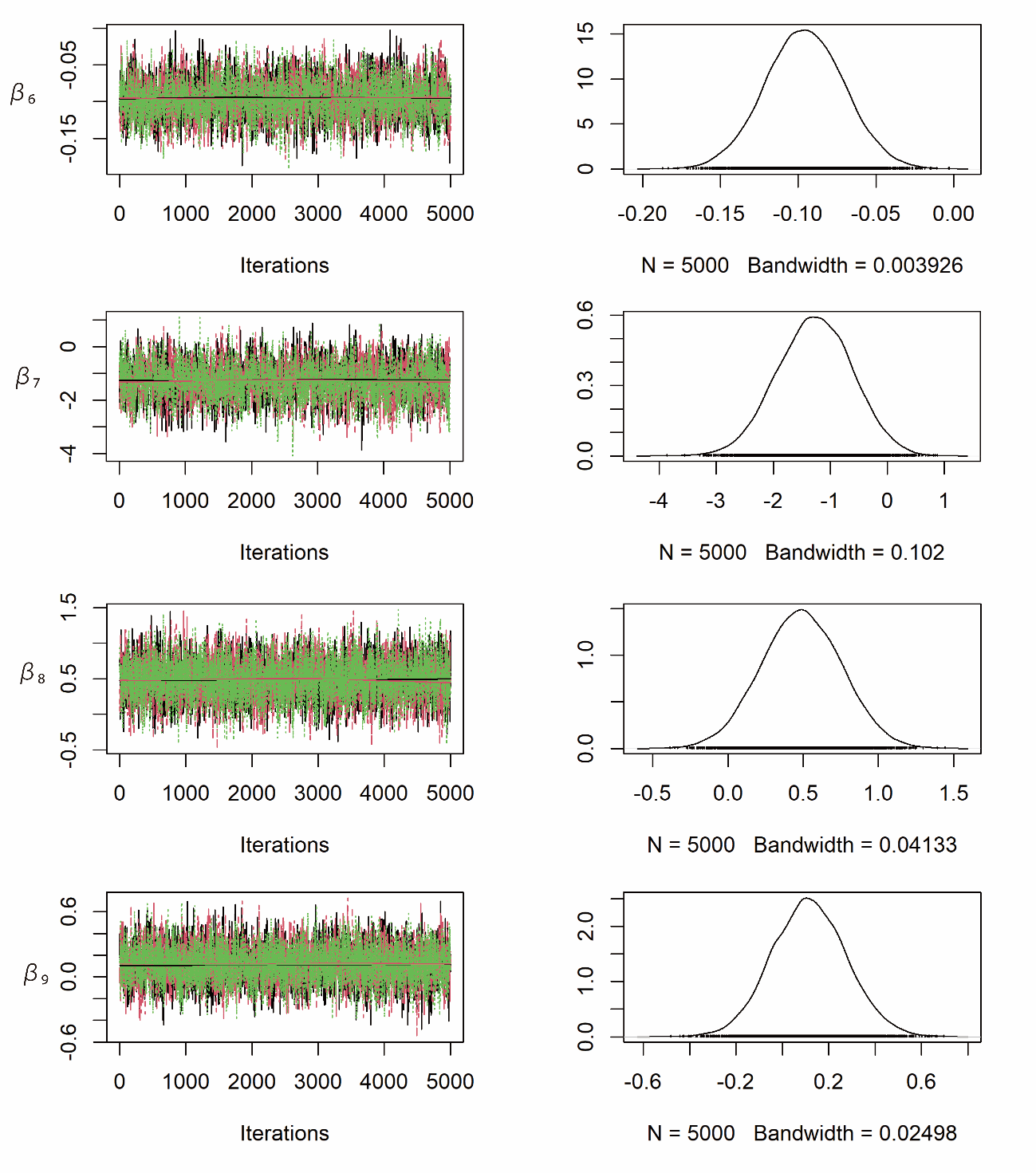


Figure S1 (continued)


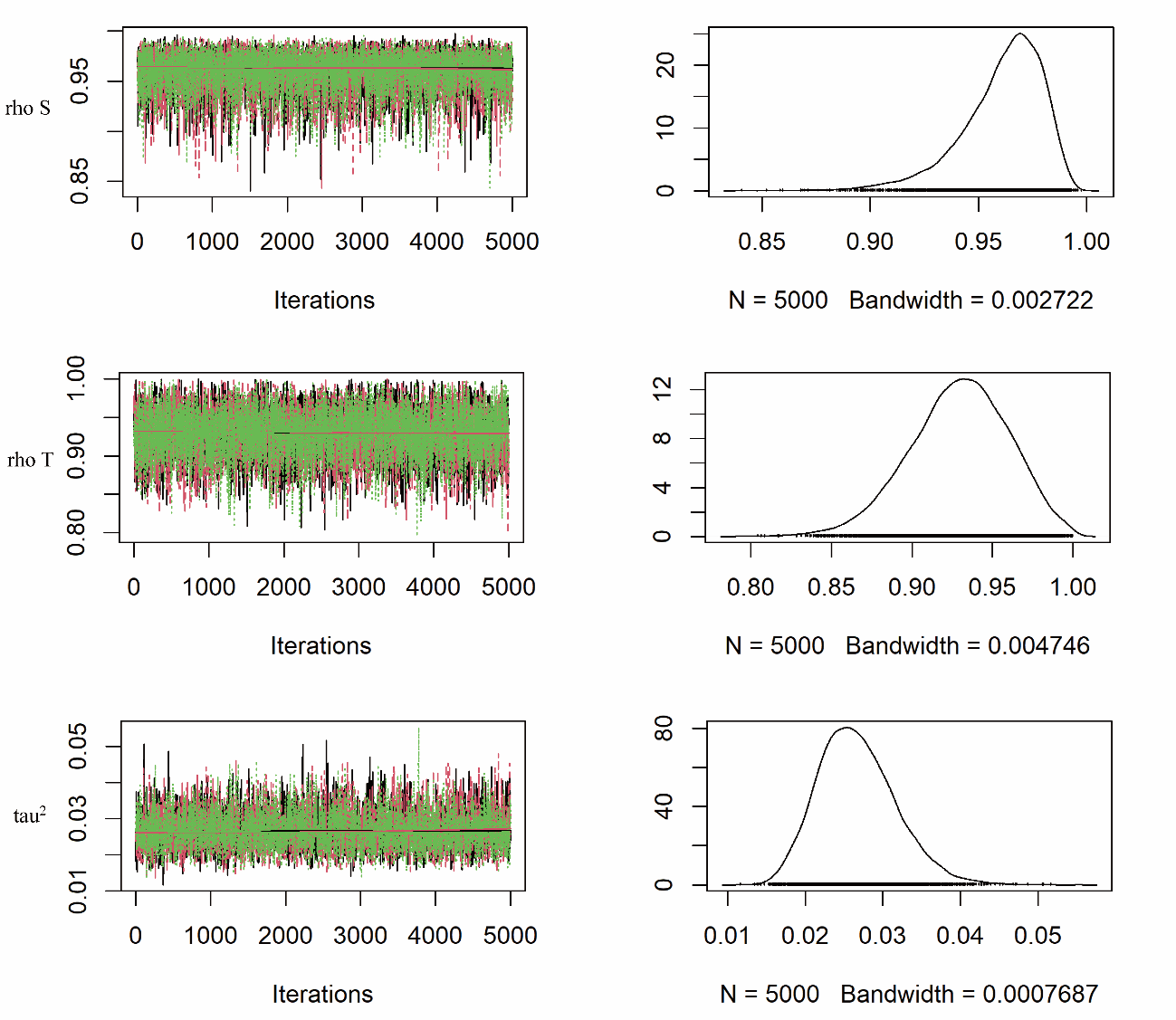


Table S1. Potential scale reduction factors of Models 1 and 2 by Gelman-Rubin.

|  | Model 1 | |
| --- | --- | --- |
|  | Point estimation | 95% CI upper bound |
| Intercept | 1.00 | 1.00 |
| Physicians | 1.00 | 1.00 |
| Hospitals | 1.00 | 1.01 |
| Clinics | 1.00 | 1.00 |
| Distance to PSC | 1.01 | 1.04 |
| Emergency hospitals | 1.00 | 1.00 |
| The ratio of people who completed college and university studies | 1.00 | 1.01 |
| The proportion of workers in secondary industries | 1.00 | 1.00 |
| The proportion of workers in tertiary industries | 1.00 | 1.01 |
| τ^2^ | 1.00 | 1.01 |
| ρ_S_ | 1.00 | 1.00 |
| ρ_T_ | 1.00 | 1.01 |

Figure S2. Trace plot of fitted value in a sub-samples.


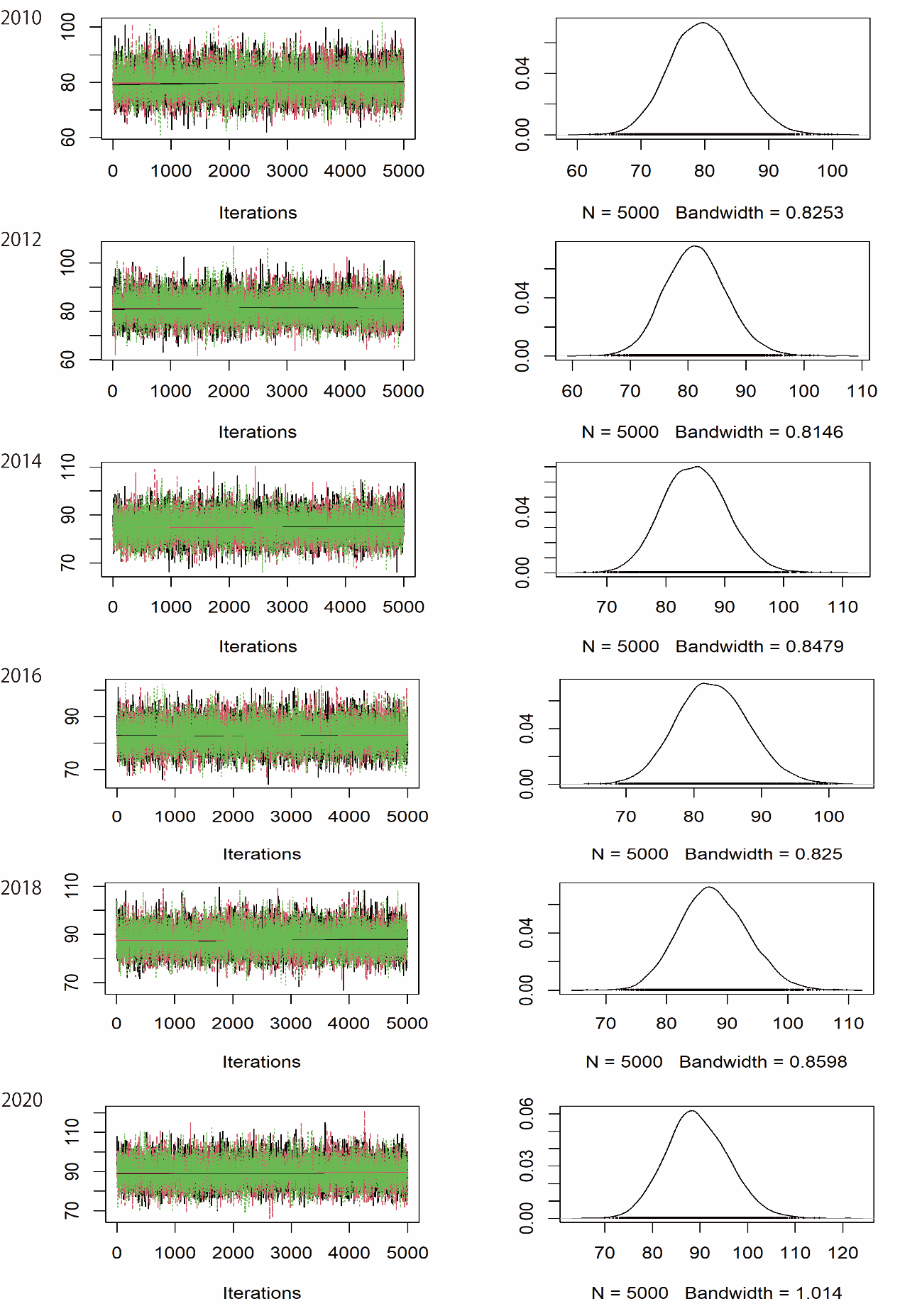

Supplement: Supplementary file 1 — Additional File 1Figure S1. Trace plot of parameters of Model 1; Table S1. Potential scale reduction factors of Models 1 and 2 by Gelman-Rubin; Figure S2. Trace plot of fitted value in a sub-samples. [file 12942_2022_316_MOESM1_ESM.docx]
