# Supplementary material for: Spatial-temporal analysis of cerebral infarction mortality in Hokkaido, Japan: an ecological study using a conditional autoregressive model
Source: Int J Health Geogr. 2022 Oct 31;21:16. doi: 10.1186/s12942-022-00316-1 (PMC9623919; doi:10.1186/s12942-022-00316-1)
Supplement: Supplementary file 2 — Additional File 2 Figure S3. Spatial pattern of cerebral infarction mortality by alternative prior (0.5, 0.005); Figure S4. Comparison of modeled SMR in three priors; Table S2. Comparison of relative risk by Sensitivity analysis for Model 1 using two distinct priors. [file 12942_2022_316_MOESM2_ESM.docx]

Figure S3. Spatial pattern of cerebral infarction mortality by alternative prior (0.5, 0.005).


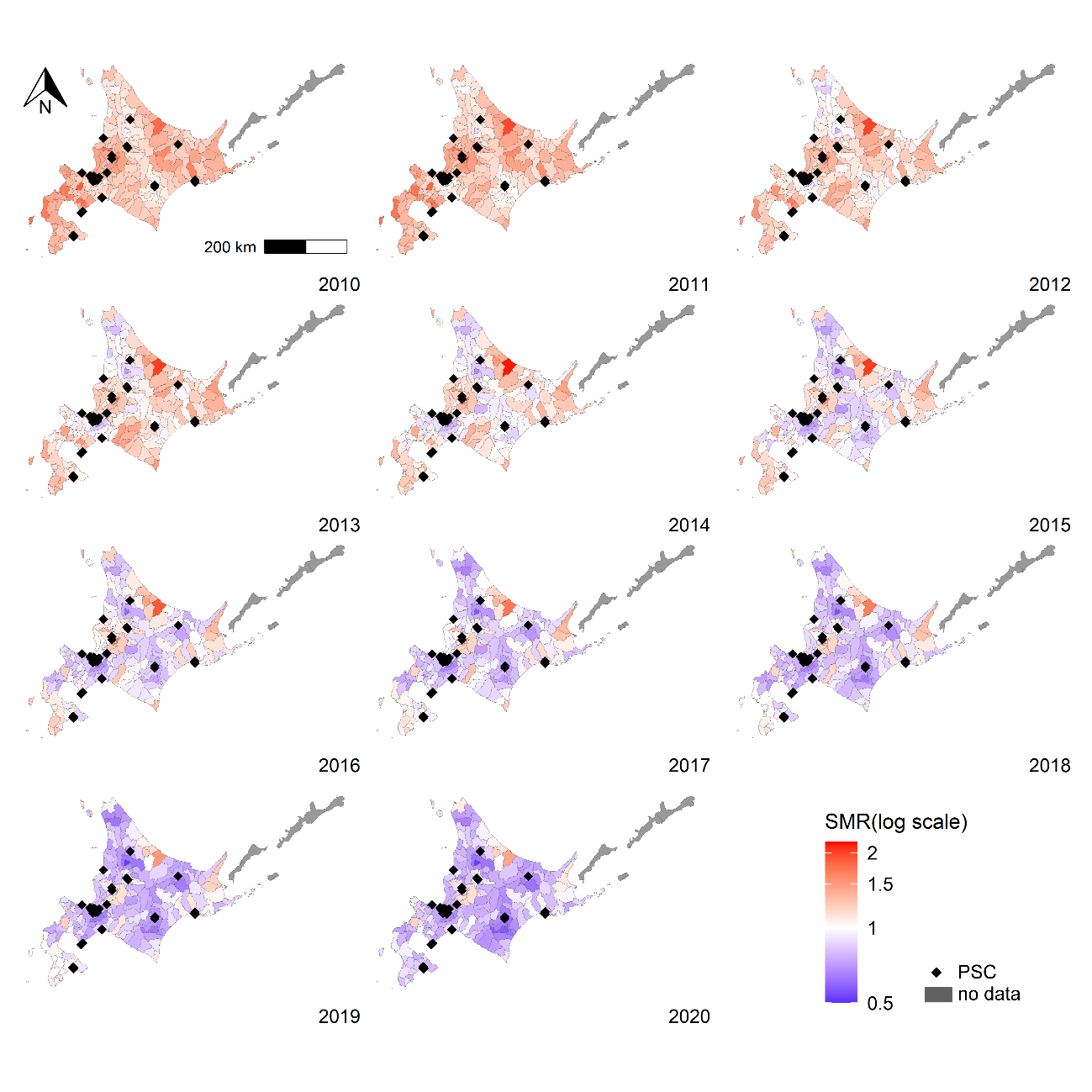


Figure S4. Spatial pattern of cerebral infarction mortality by alternative prior (1, 0.05).


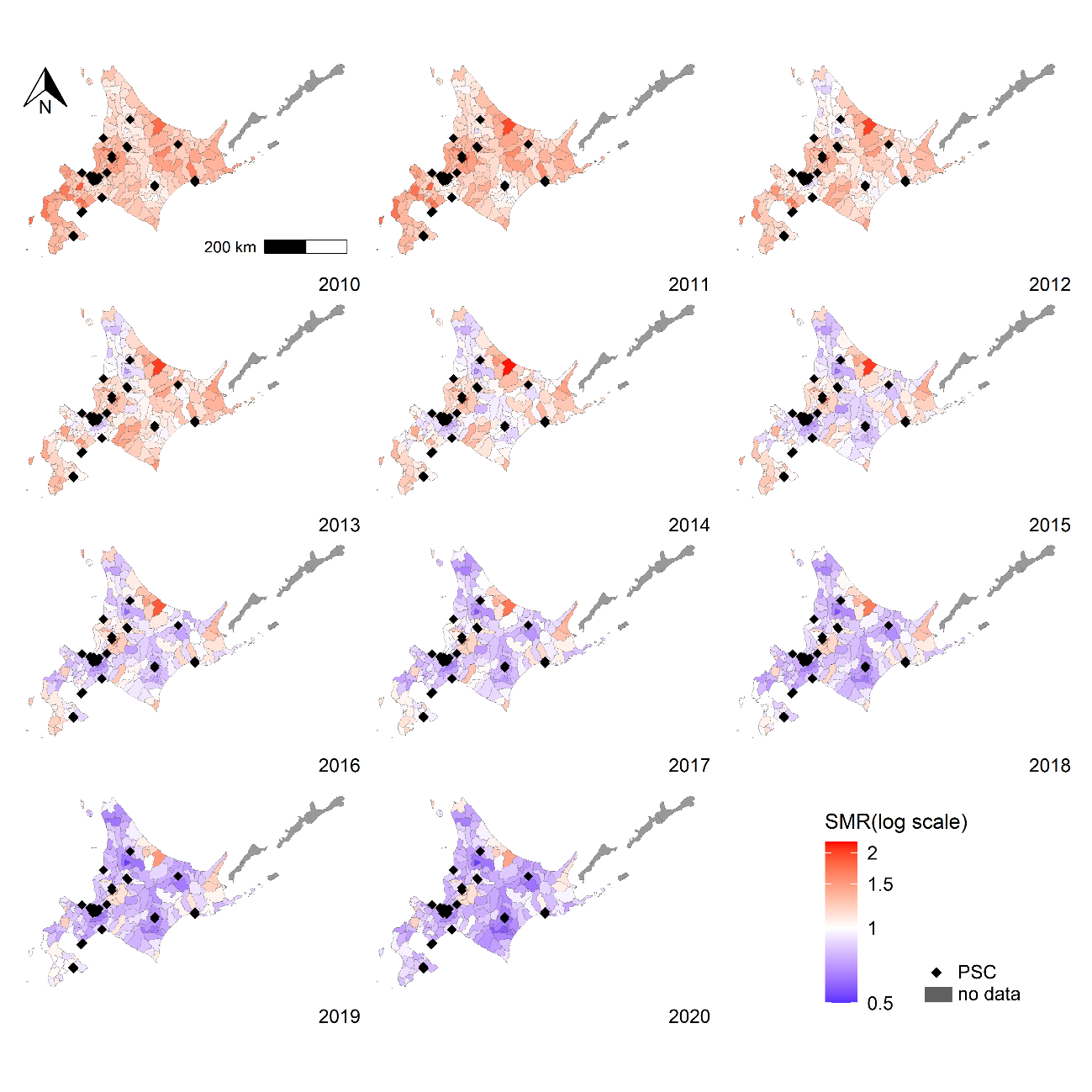


Figure S4. Comparison of modeled SMR in three priors.


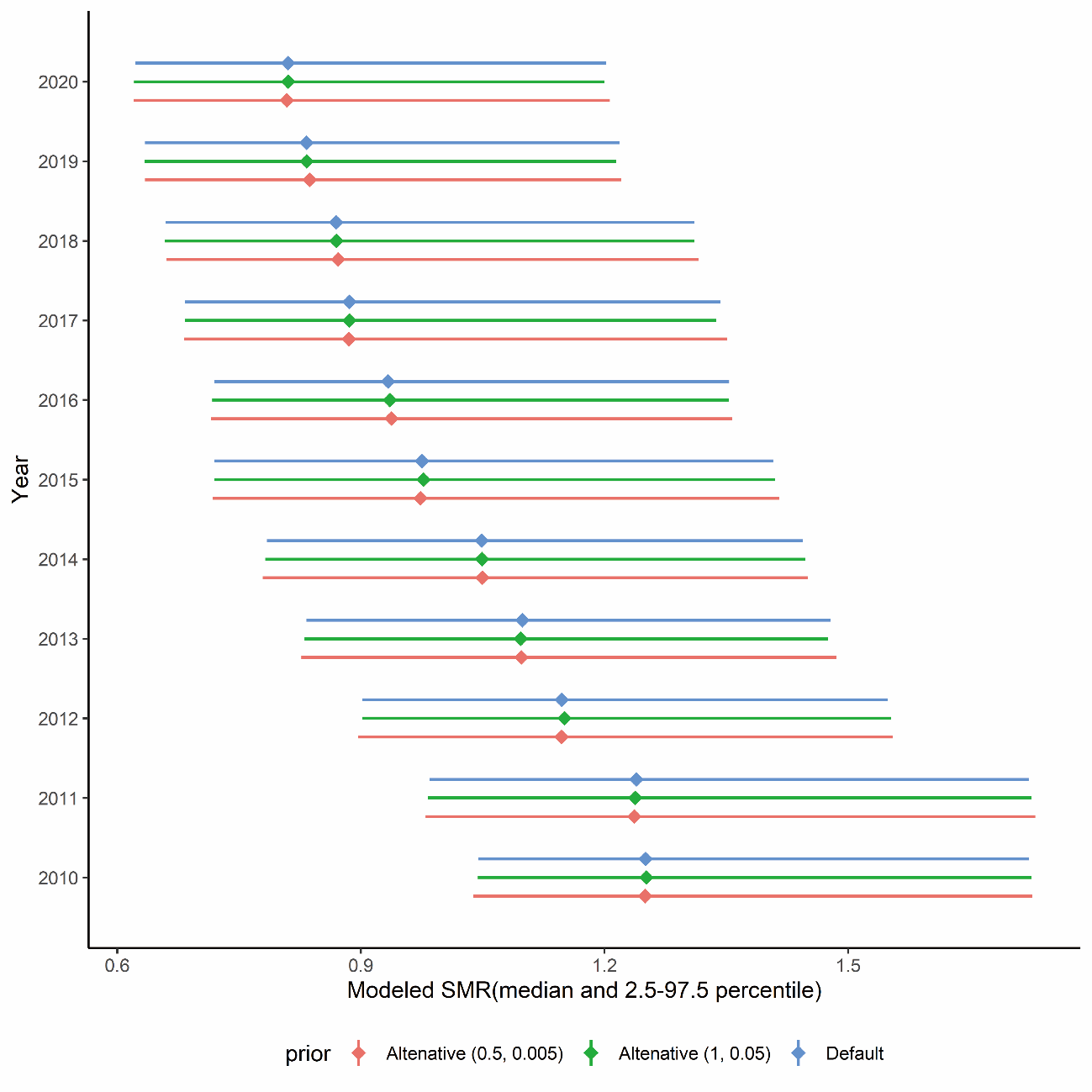


Table S2. Comparison of relative risk by Sensitivity analysis for Model 1 using two distinct priors

|  | Priors (0.5, 0.005) | | | Priors (1, 0.05) | | |
| --- | --- | --- | --- | --- | --- | --- |
|  | RR | 2.5%Cr | 97.5%Cr | RR | 2.5%Cr | 97.5%Cr |
| Physician | 1.008 | 0.988 | 1.030 | 1.009 | 0.988 | 1.030 |
| Hospitals | 1.122 | 1.067 | 1.179 | 1.122 | 1.066 | 1.179 |
| Clinics | 0.997 | 0.961 | 1.033 | 0.997 | 0.962 | 1.033 |
| Distance to PSC | 1.061 | 1.016 | 1.110 | 1.061 | 1.015 | 1.110 |
| Emergency hospitals | 0.907 | 0.863 | 0.955 | 0.907 | 0.863 | 0.954 |
| The ratio of people who completed college and university studies | 0.965 | 0.928 | 1.003 | 0.964 | 0.928 | 1.001 |
| The proportion of workers in secondary industries | 1.031 | 0.997 | 1.064 | 1.030 | 0.998 | 1.063 |
| The proportion of workers in tertiary industries | 1.012 | 0.979 | 1.047 | 1.012 | 0.979 | 1.047 |
